# Fengycins From *Bacillus amyloliquefaciens* MEP_2_18 Exhibit Antibacterial Activity by Producing Alterations on the Cell Surface of the Pathogens *Xanthomonas axonopodis* pv. *vesicatoria* and *Pseudomonas aeruginosa* PA01

**DOI:** 10.3389/fmicb.2019.03107

**Published:** 2020-01-21

**Authors:** Daniela B. Medeot, Maricruz Fernandez, Gustavo M. Morales, Edgardo Jofré

**Affiliations:** ^1^Instituto de Biotecnología Ambiental y Salud, Consejo Nacional de Investigaciones Científicas y Técnicas, Río Cuarto, Argentina; ^2^Departamento de Ciencias Naturales, Facultad de Ciencias Exactas, Físico-Químicas y Naturales, Universidad Nacional de Río Cuarto, Río Cuarto, Argentina; ^3^Departamento de Química, Facultad de Ciencias Exactas, Físico-Químicas y Naturales – Instituto de Investigaciones en Tecnologías Energéticas y Materiales Avanzados, Consejo Nacional de Investigaciones Científicas y Técnicas, Universidad Nacional de Río Cuarto, Río Cuarto, Argentina

**Keywords:** cyclic lipopeptides, atomic force microscopy, *Bacillus amyloliquefaciens*, cell surface, fengycin treatments

## Abstract

*Bacillus amyloliquefaciens* MEP_2_18 is an autochthonous bacterial isolate with antibacterial and antifungal activities against a wide range of phytopathogenic microorganisms. Cyclic lipopeptides (CLP), particularly fengycins, produced by this bacterium; are the main antimicrobial compounds responsible for the growth inhibition of phytopathogens. In this work, the CLP fraction containing fengycins with antibacterial activity was characterized by LC-ESI-MS/MS. In addition, the antibacterial activity of these fengycins was evaluated on the pathogens *Xanthomonas axonopodis* pv. *vesicatoria* (Xav), a plant pathogen causing the bacterial spot disease, and *Pseudomonas aeruginosa* PA01, an opportunistic human pathogen. *In vitro* inhibition assays showed bactericidal effects on Xav and PA01. Atomic force microscopy images revealed dramatic alterations in the bacterial surface topography in response to fengycins exposure. Cell damage was evidenced by a decrease in bacterial cell heights and the loss of intracellular content measured by potassium efflux assays. Furthermore, the viability of MRC-5 human normal lung fibroblasts was not affected by the treatment with fengycins. This study shows *in vivo* evidence on the less-known properties of fengycins as antibacterial molecules and leaves open the possibility of using this CLP as a novel antibiotic.

## Introduction

Among the notable characteristics of the members of the *Bacillus subtilis* group, which is originally composed by the species *B. subtilis, Bacillus licheniformis, Bacillus pumilus*, and *Bacillus amyloliquefaciens* and subspecies derived from them (Fan et al., 2017; Caulier et al., 2019), are the production of several bioactive molecules to control crop diseases (Fira et al., 2018; Rabbee et al., 2019) and the ability to form endospores, the resistance structures to withstand adverse environmental conditions. *B. subtilis* group strains act antagonistic to a broad range of pathogens by producing a wide diversity of secondary metabolites (Caulier et al., 2019). It is estimated that 4 to 5% of the genome of the strains belonging to the *B. subtilis* group is dedicated to the production of antimicrobial molecules, which are typically antimicrobial peptides with diverse chemical structures and moieties. Bioactive volatile metabolites and polyketides are also produced by the *B. subtilis* group (Stein, 2005; Caulier et al., 2019). These characteristics give *Bacillus subtilis* group species great competitive advantages and make these attractive microorganisms for the industrial production of biopesticides and antibiotics (Borriss, 2011; Wu et al., 2015; Fira et al., 2018; Rabbee et al., 2019).

Among the broad spectrum of bioactive secondary metabolites produced by the *Bacillus subtilis* group, the antifungal activity of cyclic lipopeptides (CLP) including the three families, surfactins, iturins and fengycins, have been widely documented (Ongena and Jacques, 2008; Raaijmakers et al., 2010; Cochrane and Vederas, 2016). In addition to antifungal properties, antiviral, anticancer, and antibacterial have also been reported (Ongena and Jacques, 2008; Wu et al., 2017; Zhao et al., 2017, 2018). The CLP exhibit others plant growth-promoting features desirable for agriculture, for example as a signal inducer of systemic resistance in plants (Ongena et al., 2005; Ongena and Jacques, 2008; Cawoy et al., 2014; Chowdhury et al., 2015). The biosynthesis of CLP is carried out by multifunctional megasynthetases called non-ribosomal peptide synthetases (NRPSs). The peptide moiety synthesized by these megaenzymes can be short, linear, cyclic, or branched-cyclic. Amino acids modifications such as epimerization, methylation or hydroxylation are frequent, giving place to a vast structural diversity of peptides produced by each *Bacillus* strain (Gao et al., 2018). Therefore, it is interesting to explore the antimicrobial activities of CLP produced by novel native *Bacillus* strains.

The antibacterial activity of CLP against several pathogens such as *Corynebacterium variabilis*, *Acinetobacter*, *Staphylococcus aureus*, *B. subtilis*, *Salmonella typhimurium*, *Pseudomonas aeruginosa*, *Enterobacter cloacae* and *Xanthomonas campestris*, among others, has been attributed to iturins and surfactins (Zhao et al., 2018). Fengycins are one of the major types of CLP produced by *Bacillus*, and activity reports have mainly been restricted to antifungal effects (Ongena and Jacques, 2008; Han et al., 2015; Wu et al., 2015; Cochrane and Vederas, 2016; Lu et al., 2016; Fira et al., 2018). For example, fengycin BS155 induces alterations at the level of the cell membrane and organelles, processes that lead to cell death in *Magnaporthe grisea* (Zhang and Sun, 2018).

Nevertheless, some authors have also reported a significant but indirect relationship between the antibacterial activity and the production of fengycins. Such antibacterial activity was only evidenced after the modification of fengycin molecules by self-assembly (Roy et al., 2013). Previously, we demonstrated that *B. amyloliquefaciens* MEP_2_18 (MEP_2_18), a plant growth-promoting rhizobacterium (PGPR), produces fengycins A and B when grown in an optimized lipopeptides production medium. These fengycins exhibited an increased antibacterial activity against *Xanthomonas axonopodis* pv. *vesicatoria* (Xav), the phytopathogenic bacterium causing the widespread and harmful bacterial spot disease on tomato plants (*Solanum lycopersicum*) in comparison with those obtained in the unmodified culture medium (Medeot et al., 2017).

The effect of fengycins on the bacterial cell is poorly documented. Most studies show that fengycins are inserted into model biomembranes, due to its amphiphilic characteristics, establishing fengycin-rich domains which constitute permeabilization sites that result in a leaky target membrane (Deleu et al., 2008; González-Jaramillo et al., 2017).

The aim of this work was to study the effect of fengycins produced by MEP_2_18 on two pathogenic bacteria by using atomic force microscope (AFM) and to evaluate the cytotoxic effect of this CLP on human normal lung fibroblasts. The pathogenic bacteria selected for the experiments were Xav, as phytopathogen model, and *P. aeruginosa* PA01, an opportunistic pathogen of growing clinical relevance. Xav is a phytopathogen that has acquired resistance to the most commonly used control methods. The emergence of copper-tolerant *Xanthomonas* strains constitutes a serious problem for farmers (Potnis et al., 2015; Strayer-Scherer et al., 2018). *P. aeruginosa* is an opportunistic pathogen that, due to its metabolic versatility, is able to colonize several niches, being a serious problem in hospitalized patients and a leading cause of morbidity and mortality in patients with cystic fibrosis or immunocompromised. The control of infections caused by *P. aeruginosa* is increasingly difficult due to its notorious ability to resist antibiotics either through intrinsic or acquired resistance mechanisms. The finding and development of alternative therapeutic strategies for controlling infections produced by *P. aeruginosa* are increasingly demanded (Pang et al., 2019).

The AFM technique allowed us to visualize, at the single cellular level, that fengycins induced changes on the cell topography which probably are associated with cell death. The confirmation of the damage to the cellular envelope caused by fengycins was obtained from the data of potassium efflux assays. Furthermore, the promising antibiotic properties of fengycins are supported by the non-cytotoxic effects since the viability of human normal lung fibroblasts (cell line MRC-5) was not affected by fengycins treatment.

## Materials and Methods

### Microorganisms and Growth Conditions

The MEP_2_18 strain is an autochthonous bacterium isolated from a saline soil of the south of the Córdoba province, Argentina. This strain possesses biocontrol properties (Príncipe et al., 2007; Alvarez et al., 2012). The MEP_2_18 was grown in Lysogeny broth (LB) or in Modified Medium Optimal for Lipopeptide Production (MMOLP) (Medeot et al., 2017) for 48 h at 30°C and 150 rev min^–1^. Xav, the phytopathogenic bacterium causing the bacterial spot disease on pepper (*Capsicum* spp.) and tomato (*Lycopersicon* spp.) plants (Thieme et al., 2005), was routinely grown for 24 h at 30°C on nutrient agar (NA) plates or nutrient broth. *Pseudomonas aeruginosa* PA01 (ATCC 15692), a well-known and metabolically versatile opportunistic human pathogen, was routinely grown for 24 h at 37°C on LB agar plates. All strains were stored at −80°C in saline buffer (0.7% Na_2_HPO_4_, 0.3% KH_2_PO_4_, and 0.5% NaCl) plus 20% glycerol.

### Isolation of Cyclic Lipopeptides and Fengycin Purification

Cyclic lipopeptides from MEP_2_18 were precipitated from the cell free culture supernatant following acidification with HCl (12 N) until pH = 2,0 (Vater et al., 2002; Kim et al., 2004; Medeot et al., 2017). Precipitated CLP were suspended in 100% methanol, filtered through a 0.22 μm nylon membrane, and injected onto a high-performance liquid chromatography (HPLC) (Infinity LC Grad, Agilent) column (Analytical Zorbax C18, 4.6 mm × 150 mm, Agilent). The temperature was maintained at 37°C. Separation was achieved by using a mixture of solvents consisting of acetonitrile (solvent A) and water acidified with 0.1% formic acid (solvent B) with a flow rate of 1 mL min^–1^. For each run, 100 μl of sample was injected into the HPLC column and eluted using the conditions described in Medeot et al. (2017).

The peaks with a retention time of 27.4 min, corresponding to fengycins (Medeot et al., 2017), from several HPLC runnings were collected, lyophilized and the powder was weighed and suspended in 100% methanol at final concentrations of 1 and 10 mg mL^–1^. These solutions were used as stocks of fengycins.

### Mass Spectrometry

Fengycins stock (1 mg mL^–1^) was submitted to the Center for Chemical and Biological Studies Maldi Tof Spectrometry (CEQUIBIEM, University of Buenos Aires, Argentina) for spectrometric analysis. Fengycins were purified with ZipTips C18 and analyzed by nanoHPLC (Thermo Scientific, EASY-nLC 1000) equipped with a reverse-phase C18 column (Easy-Spray ColumnPepMap RSLC, P/N ES803, 2 μm particle size, 75 μm × 500 mm, Thermo Scientific) at 35°C. A two component solvent system was used: solvent A is water (HPLC grade) acidified with 0.1% formic acid and solvent B is acetonitrile with 0.1% formic acid. For each run, 4 μl of sample was injected into a nanoHPLC system and eluted using the following gradient (% A:B v/v): injection start (95:5), 5 min isocratic (95:5), then an increasing gradient of solvent B to 50% through 80 min, then an increasing gradient of solvent B to 80% through 5 min and finally 10 min isocratic (20:80). The elution program used a flow rate of 200 nL min^–1^.

For ESI-MS/MS experiments the spectra were recorded in positive ion mode using a mass spectrometer (Thermo Scientific, Q-Exactive) with a High Collision Dissociation (HCD) cell and an Orbitrap analyzer. The ionization of the samples was done by electrospray (Thermo Scientific EASY-SPRAY. Voltage: 2,25 kV). The 10 most abundant ions of each scan were subjected to MS/MS analysis with a dynamic exclusion range. The analysis of the data was performed with the Xcalibur 3.1 software (Thermo Scientific).

### Analysis of Antibacterial Activity of Fengycin

The antibacterial activity of fengycins against Xav and PA01 was tested on NA and LB agar plates respectively, by using the disk diffusion method as described by Medeot et al. (2017). Briefly, an aliquot from a culture of Xav and PA01 (grown until OD_600_ = 0.3–0.4, the logarithmic phase of growth) was spread on a plate. Different concentrations (6.25, 12.5, and 25 μg mL^–1^ for Xav and 25, 50, 100, and 200 μg mL^–1^ for PA01) of fengycins (contained in a final volume of 10 μL) were deposited on sterile paper disks. The imbibed paper disks were left for 10 min under the sterile airflow to allow methanol evaporation, and then they were deposited onto the plate. After incubation for 24 h at 30°C, inhibition zones were visualized and the diameter of the zone formed around each paper disk was recorded. Control consisted of paper disks imbibed with 10 μL of methanol. The assay was performed in triplicate.

### Determination of Minimal Inhibitory Concentration

The minimum inhibitory concentration (MIC) of fengycins on Xav and PA01 was determined by the microtiter plate dilution assay (Baindara et al., 2013). Xav and PA01 were grown until OD_600_ = 0.3–0.4 in nutritive broth or LB, respectively. Aqueous solutions of 0, 1.5, 3.12, 6.25, 12.5, 25, 50, 100, and 200 μg ml^–1^ of fengycins were used. The lowest concentration of fengycins that inhibited the growth of the bacteria and did not show any increase in the OD600 after 48 h of incubation was considered as the MIC. The assay was performed in triplicate.

### Cell Viability Determination

#### Cell Culture

MRC-5 human normal lung fibroblasts were cultured as previously described (Lamberti et al., 2019). Briefly, they were grown in complete medium DMEM (Dulbecco’s modified Eagle medium high glucose 1X, Gibco) supplemented with 10% v/v fetal bovine serum (FBS) (PAA Laboratories), 1% v/v glutamine (GlutaMAXTM 100X Gibco), 1% v/v antibiotic (Penicillin 10,000 units mL^–1^ – streptomycin 10,000 μg mL^–1^ Gibco) and 1% v/v of sodium pyruvate 100 mM (Gibco), in 5% CO_2_ and 95% air at 37°C in a humidified incubator. Stock cultures were stored in liquid nitrogen and used for experimentation within 5 to 7 passages.

#### Cell Treatment

Exponential-phase MRC-5 cells at a density of 5 × 10^4^ cells mL^–1^ were plated onto 96-well plates, using 100 μl per well in complete medium. Following an overnight incubation to allow attachment, fengycins (100–200 μg/ml) were added to each well and incubated for 24 h (37°C, 5% CO_2_) in complete medium. Control cells were treated with vehicle alone (phosphate buffer saline). The assay was performed in triplicate.

#### Cell Viability Quantification

Cell viability was evaluated by 1-(4,5-dimethylthiazol-2-yl)-3,5-diphenylformazan (MTT) assay, which is reduced by mitochondrial dehydrogenases of viable cells to non-water soluble violet formazan crystals, as previously described (Lamberti et al., 2013, 2018). Briefly, MTT solution (5 mg mL^–1^ in phosphate buffer saline) was added for 3 h (dilution rate: 1/10). Then, DMSO was added to lyse the cells and solubilize the precipitated formazan product. Optical density of the formazan salt was read at 540 nm using ELISA reader plate (Thermo Scientific, Multiskan FC). The assay was performed in triplicate.

### Atomic Force Microscopy of Bacterial Cells Treated With Fengycin

Cells of Xav and PA01 were obtained after centrifugation (3500 × *g* for 2 min) of cultures grown until OD_600_ = 0.5. Cells were washed three times and suspended in water. Cell suspensions were treated with fengycins (dissolved in water) at final concentrations of 12.5, 25, 50, and 75 μg mL^–1^ for Xav, and 100 and 200 μg mL^–1^ for PA01. The suspensions were incubated for 90 min for Xav and 6 h for PA01 at 28°C and 150 rpm, and later washed twice with water. The control experiment consisted of the same treatment but replacing the fengycins by water. The assay was performed in triplicate.

Afterward, treated bacterial cells were immobilized electrostatically by depositing 15 μL of the bacterial suspension over polyethyleneimine coated glass slides (Dufrêne, 2008). After 30 min of incubation at 28°C, the slides were washed with water, dried and mounted for observation. Topographic images were obtained with an AFM (Agilent, Technologies, SPM model 5500) in air and working in acoustic AC mode (Fernandez et al., 2017). The experiments were performed using AFM probes (Micromasch, HQ:XSC11/A1 BS) with a cantilever resonance frequency and force constant of 155 kHz and 7 N/m respectively. The bacterial height analysis was performed by measuring 70 individual cells per treatment, using statistical tools and modules offered in Gwyddion v2.39^1^. Analysis of variance followed by Tukey test was carried out by using software R^2^. In all cases, *p* ≤ 0.05 was considered as significance level.

### Potassium Ion Efflux

Cells of Xav, grown in nutrient broth at 30°C until OD_600_ = 0.5, were collected by centrifugation (10,000 × *g* for 15 min) and washed three times with water. After resuspension in water, at the original volume, each sample was split into two. One of them was supplemented with fengycins at the MIC value (25 μg mL^–1^) and another three times higher to ensure the total cell lysis (75 μg mL^–1^) and the other one with water (negative control). After 90 min of treatment, aliquots (1 mL) of each cell suspensions were centrifuged at 13,000 × *g* for 5 min and the supernatants were filtered by using a 0.2 μm pore size filter and transferred to new tubes. Positive control consisted of cell suspensions boiled for 10 min to release intracellular K^+^ ions (Cushnie and Lamb, 2005). The concentration of K^+^ ions was determined in the supernatants using a single-channel flame photometer (Digital Flame Analyzer Cole-Parmer). Calibration curves were obtained with potassium standards in the range of 1–20 ppm K^+^ mL^–1^. The results informed are the average of three different experiments with three replicates.

## Results

### LC-ESI-MS/MS Analysis of Fengycins From *B. amyloliquefaciens* MEP_2_18

**FIGURE 1:**
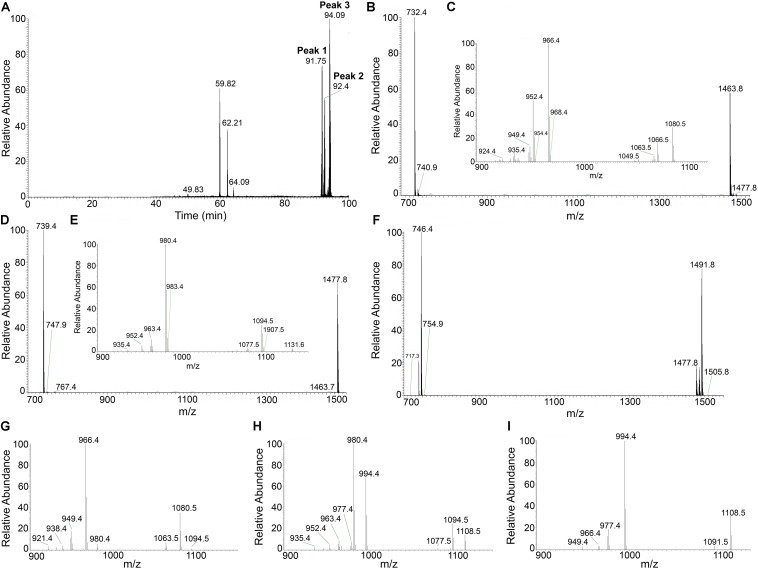
LC-ESI-MS/MS analysis of fengycins from *Bacillus amyloliquefaciens* MEP_2_18. Elution profile of fengycins **(A)**, obtained from a HPLC-purified peak after separation of CLP from *B. amyloliquefaciens* MEP_2_18, using nanoHPLC technology (Thermo Scientific, EASY-nLC 1000) equipped with a reverse-phase C18 column (Easy-Spray ColumnPepMap RSLC, P/N ES803, 2 μm particle size, 75 μm × 500 mm, Thermo Scientific). ESI-MS/MS spectra of protonated fengycin ions *m/z* 1463.8 **(B)** obtained from peak 1, the spectrum of precursor ion of *m/z* 1463.8 **(C)**; *m/z* 1477.8 **(D)** obtained from peak 2, the spectrum of precursor ion of *m/z* 1477.8 **(E)**; and ions *m/z* 1477.8, 1491.8, and 1505.8 **(F)** obtained from peak 3, the spectrum of precursor ion of *m/z* 1477.8 **(G)**, 1491.8 **(H)**, and 1505.8 **(I)** were acquired in a mass spectrometer (Thermo Scientific, Q-Exactive) with a high collision dissociation (HCD) cell and an Orbitrap analyzer.

Previously, we reported that changes in the sources and ratios of C and N in the culture medium can modulate the production of CLP in MEP_2_18. Specifically, MMOLP medium enhanced the production of different isoforms of fengycins A and B (Medeot et al., 2017). To accurately determine the variants of fengycins produced by the strain MEP_2_18 in MMOLP, the HPLC-purified fraction eluted at 27.4 min and containing the fengycins was subjected to liquid chromatography–electrospray ionization mass/mass spectrometry (LC/ESI-MS/MS) analysis. The LC profile shows three peaks, at the retention times where fengycins elute (Figure 1A). Fengycins were identified according to the typical mass values of these compounds (1400–1500 Da) (Monaci et al., 2016). Mass spectra of peaks 1, 2, and 3 from LC (Figure 1A) displayed peaks at *m/z* 1463.8 (Figure 1B), 1477.8 (Figure 1D), 1477.8, 1491.8, and 1505.8 (Figure 1F), respectively, and they represent different fengycins homologs. From each ion, selected as the precursor ion, an additional fragmentation was performed and the resultant ESI-MS/MS spectrum was analyzed. Product ions at *m/z* 966 and 1080 were found in the MS/MS spectra of precursor ions at *m/z* 1463.8 and 1477.8 (Figures 1C,G) while product ions at *m/z* 994 and 1108 were observed in MS/MS spectra of precursor ions at *m/z* 1491.8 and 1505.8 (Figures 1H,I). Product ions at *m/z* 1080 and 966 correspond to fengycin A and are generated by the neutral losses of fatty acid –Glu and fatty acid –Glu–Orn, respectively, from the N-terminus segment of fengycin A (Wang et al., 2004). Similarly, product ions at *m/z* 1108 and 994 also indicate neutral losses of fatty acid –Glu and fatty acid –Glu–Orn, but they are 28 Da heavier than those ions of fengycin A. The observed mass difference indicates the substitution of Ala for Val at position 6 in the lactone ring and therefore homologs at *m/z* 1491.8 and 1505.8 are fengycin B (Wang et al., 2004).

The ESI-MS/MS spectrum of LC peak 2 (*m/z* 1477.8) corresponds to fengycin (Figure 1D) and displays two product ions at *m/z* 980.4 and 1094.5 (Figure 1E). According to the *m/z* values reported by Li et al. (2012) and Pathak et al. (2012), these product ions correspond to fengycins C, D (or B2) and/or S.

Based on the results obtained, Table 1 summarizes the correct assignment of fengycins produced by *B. amyloliquefaciens* MEP_2_18 in MMOLP medium, detected in the HPLC fraction that eluted at 27.4 min.

**TABLE 1:** LC/ESI-MS/MS assignment of protonated fengycins homologs contained in the 27.4 min fraction from *Bacillus amyloliquefaciens* MEP_2_18 strain.

**Retention time**	**Mass peak (*m/z*)**	**Typical product ions**		**Amino acid at position 6**	**Amino acid at position 10**	**Assignment**
91.75	1463.8	1066.5	952.4	Ala	Val	C-16 fengycin A2
		1080.5	966.4	Ala	Ile	C-17 fengycin A
92.43	1477.8	1094.5	980.4	Ala/Val/Ile/Leu	Ile/Val	C-16 fengycin C, B2, S
94.00	1477.8	1080.5	966.4	Ala	Ile	C-17 fengycin A
94.13	1491.8	1108.5	994.4	Val	Ile	C-16 fengycin B
94.56	1505.8	1108.5	994.4	Val	Ile	C-17 fengycin B

### Antibacterial Activity of C16-C17 Fengycins From *B. amyloliquefaciens* MEP_2_18 on *Xanthomonas axonopodis* pv. *vesicatoria* and *Pseudomonas aeruginosa* PA01

**FIGURE 2:**
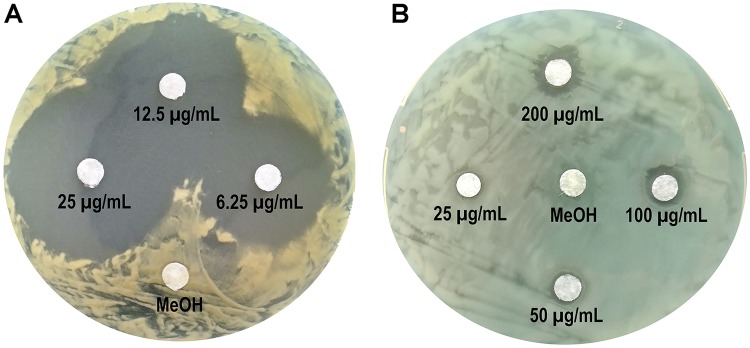
Antibacterial activity of fengycins from *B. amyloliquefaciens* MEP_2_18 against *Xanthomonas axonopodis* pv. *vesicatoria* **(A)** and *Pseudomonas aeruginosa* PA01 **(B)**. Paper disks were imbibed with 10 μL of fengycins (at 6.25, 12.5 and 25 μg mL^–1^ for Xav, and 25, 50, 100, and 200 μg mL^–1^ for PA01) from MEP_2_18, and 10 μL of methanol (MeOH) as a control.

Considering the fact that fengycins produced by MEP_2_18 displayed a strong antibacterial activity against Xav, we assessed whether this CLP could also be effective against PA01, a typical human pathogen model. Thus, the antibacterial activity against Xav and PA01 of C16-C17 fengycins, hereafter fengycins, was evaluated by determining the MIC and the bacterial growth inhibition employing the disk diffusion method. Fengycins exhibited antibacterial activity against Xav and PA01 (Figure 2). The most susceptible bacterial strain to these fengycins was Xav, showing strong inhibition halos at the lowest concentration of fengycins assayed (6.25 μg mL^–1^) (Figure 2A). PA01 was also inhibited by fengycins although at higher concentrations (200 μg mL^–1^) (Figure 2B). The MIC values were similarly different, with that for Xav (MIC = 25 μg mL^–1^) being eight times lower than for PA01 (MIC = 200 μg mL^–1^) (data not shown). Disks in-bedded in methanol (used for making fengycin suspensions), had no inhibitory effects on Xav or PA01. These results demonstrated the antibacterial activity of fengycins against two different bacteria: Xav, a plant pathogen, and PA01, an opportunistic human pathogen.

### Determination of Toxicity of Fengycins on Human Cells

**FIGURE 3:**
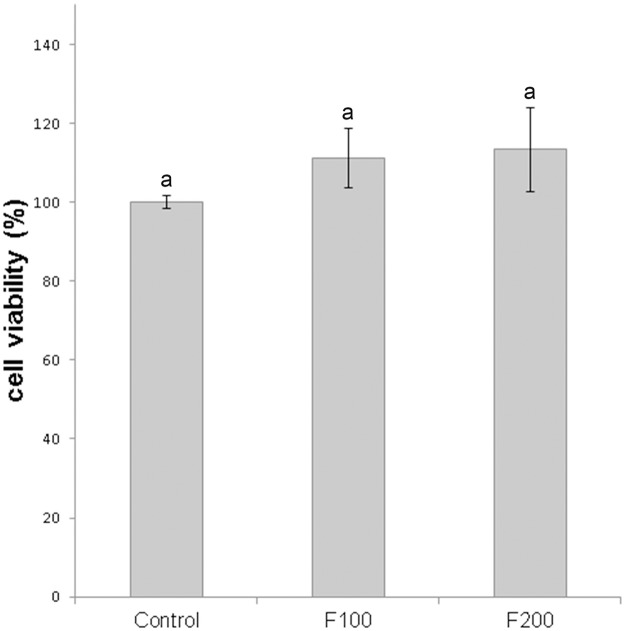
Effect of fengycins on cell viability of MRC-5 human normal lung fibroblasts. The cells were treated with 0, 100, and 200 μg mL^–1^ of fengycins (control, F100 and F200, respectively) for 24 h and then the cell viability was evaluated by MTT assay. The cell viability for the control (without fengycins), was considered as 100%. Values are the mean ± SD of three independent experiments with three biological replicates per treatment. Different letters indicate a statistically significant difference (*p* ≤ 0.05).

In order to test the toxicity of fengycins on mammal cells, the viability of human normal lung fibroblasts (cell line MRC-5) was determined after treatment with fengycins at 100 and 200 μg mL^–1^ by using the 1-(4,5-dimethylthiazol-2-yl)-3,5-diphenylformazan (MTT) assay. The viability of fibroblasts treated with fengycins did not show any significant change (*p* ≤ 0.05) compared with the control treatment (phosphate buffered saline), suggesting that fengycins are not cytotoxic at the concentrations tested (Figure 3).

### Changes in Xav Cell Topography After Treatment With Fengycins Observed by AFM

**FIGURE 4:**
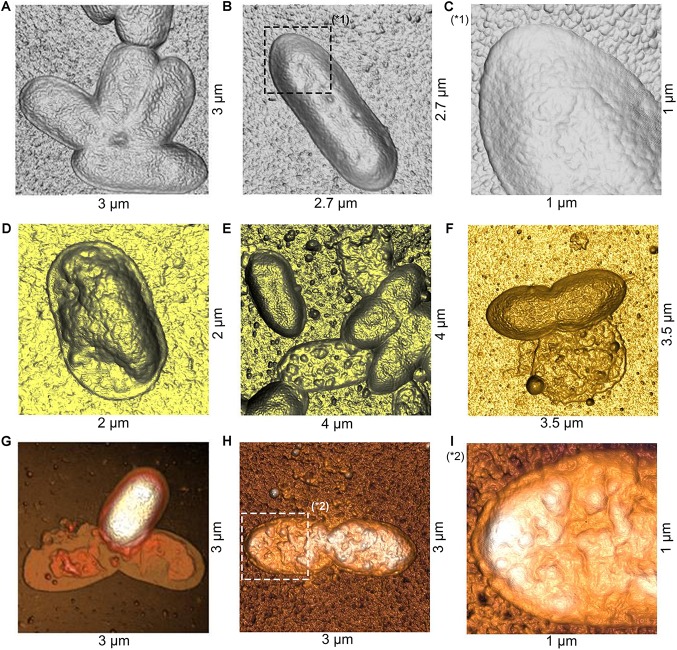
Atomic force microscopy (AFM) images of *Xanthomonas axonopodis* pv. *vesicatoria* (Xav) cells after fengycins exposure. Topography of untreated cells **(A,B)**. Cells topography following fengycins treatments with 12.5 **(D)**, 25 **(E),** 50 **(F),** and 75 **(G,H)** μg mL^–1^ for 90 min. The star symbols (^∗^) reveal zoom area of the figures **(B,C^∗^1)** and **(H,I^∗^2)**.

To study possible effects of fengycins on Xav cells at micro- or nano-scale, bacteria were treated with fengycins or water (control), and the bacterial cell topography was analyzed by using AFM. AFM is an extremely useful tool for analyzing the three-dimensional topography of biological samples, including bacteria. The technique allows the characterization of the bacterial cell surface producing high resolution topographical imaging with minimal sample disruption (Müller and Dufrêne, 2011). Cell suspensions of Xav were treated with 12.5, 25, 50, or 75 μg mL^–1^ of fengycins for 90 min. Control experiments were performed by exposing Xav to water. Representative AFM images from both control and fengycins treated Xav cells are shown in Figure 4. Control Xav cells presented a typical rod shape with a uniform surface topography (Figures 4A,B). In addition, a high-resolution image shows a relatively uniform and smooth cell surface (Figure 4C).

In comparison, clear disturbances in the cell morphology are observed in cells exposed to 12.5 μg mL^–1^ of fengycins (Figure 4D). At higher fengycins concentrations (25 and 50 μg mL^–1^), the perturbations observed on the cell surface are more evident. In some cases, only the cell boundaries with some protruding granules are observed, which gives to the cells an empty and irregular appearance. Probably, this could represent the topography of cellular debris that remains after the action of fengycins (Figure 4E). In other cases, the collapse of the whole cell was evident (Figure 4F).

Total cell damage is observed at the highest concentration of fengycins assayed (75 μg mL^–1^) (Figures 4G,H). High-resolution images of the cell surface show severe damage triggered by fengycins exposure (Figure 4I) in comparison with those of the control cells (Figure 4C). Thus, the topographical changes observed in the bacterial cells become more pronounced as the concentration of fengycins used in the treatment increases. Moreover, a clean substrate surface is observed as background around the untreated cells (Figures 4A–C). In contrast, small granules or aggregates are observed on the polymeric support close to the fengycins-treated cells (Figures 4D–I). This behavior is concomitant with the damage of cell integrity, suggesting that the aggregates could correspond to intracellular content and/or cellular debris.

### Decreasing of Xav Cell Height by Fengycins Exposure

**FIGURE 5:**
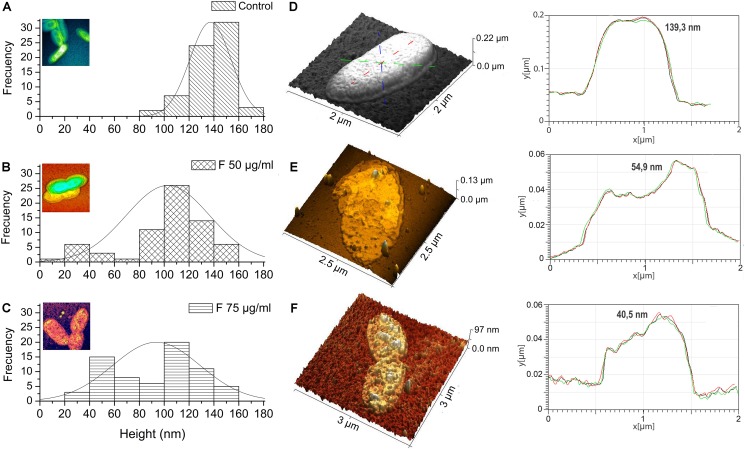
Height distribution of *Xanthomonas axonopodis* pv. *vesicatoria* (Xav) cells measured by AFM. Fengycins treatment with 50 **(B)** or 75 μg mL^–1^ **(C)** resulted in a significant decrease (*p* ≤ 0.05) in the height (across the whole cell) of 70 individual Xav cells in comparison with the untreated cells **(A)**. 3D AFM images showing changes in cell surface topography quantified using a cell height transect line across similar regions of the Xav cells and a change in cell topography and surface convolution in x, y, and z directions **(D–F)**.

Changes in the dimensions of the bacteria were quantified by measuring, from the AFM topography images (Figures 5D–F), the cellular height of 70 bacteria per treatment. The analysis of height distribution in the histogram shows that the most common frequency of observation for the untreated cells is found between 120 and 160 nm (Figure 5A). However, when treatments with fengycins were applied, the frequencies decreased to 120 nm (Figures 5B,C). In fact, compared with average height of the control cells (137 nm), a significant decrease (*p* ≤ 0.05) is observed after treatments with both 50 and 75 μg mL^–1^ of fengycins (102 and 93 nm, respectively). No significant changes were observed in the height distribution measured between the two concentrations of fengycins assayed. Therefore, the height reduction observed in cells after the treatment with fengycins suggests shrinkage in the volume of Xav cells, probably due to loss of intracellular materials by cells collapse.

**FIGURE 6:**
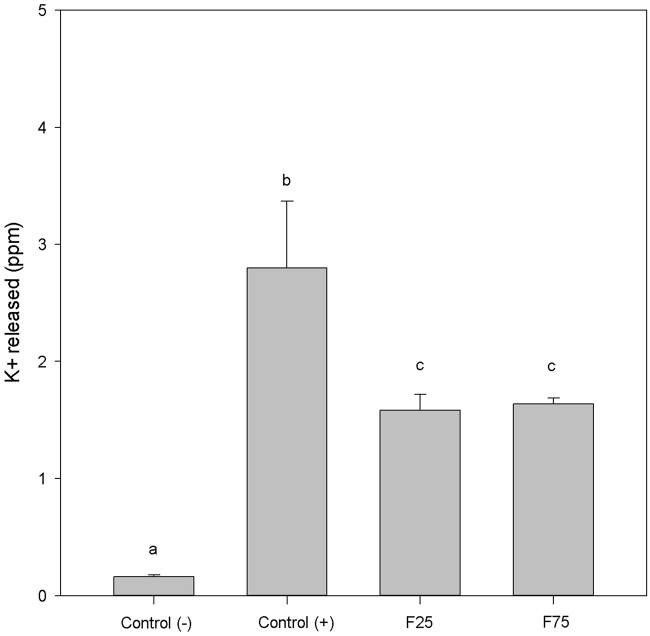
Potassium efflux of *Xanthomonas axonopodis* pv. *vesicatoria* cells treated with 25 and 75 μg mL^–1^ of fengycins for 90 min. Positive control consisted of cell suspensions boiled for 10 min to release intracellular K^+^ ions. The graph shows the means ± SE of three independent experiments with three replicates per experiment. Different letters indicate a statistically significant difference (*p* ≤ 0.05).

Potassium efflux measurement was performed to determine if the height decrease of Xav cells treated with fengycins correlated with damage in the cell envelope and the release of intracellular content. The treatment of Xav cells with fengycins at two concentrations: the MIC value (25 μg mL^–1^) and another three times higher to ensure the total cell lysis (75 μg mL^–1^), caused a significant efflux of potassium ions from Xav cells (1.58 and 1.63 ppm respectively) compared with the negative control (0.16 ppm) where water replaced the fengycins (Figure 6). The positive control, consisting of Xav cells boiled at 100°C for 10 min (total lysis), released all the intracellular potassium content (2.80 ppm).

### Fengycins Exposure Also Affects the Cell Height and Topography of the Opportunistic Pathogen *Pseudomonas aeruginosa* PA01

**FIGURE 7:**
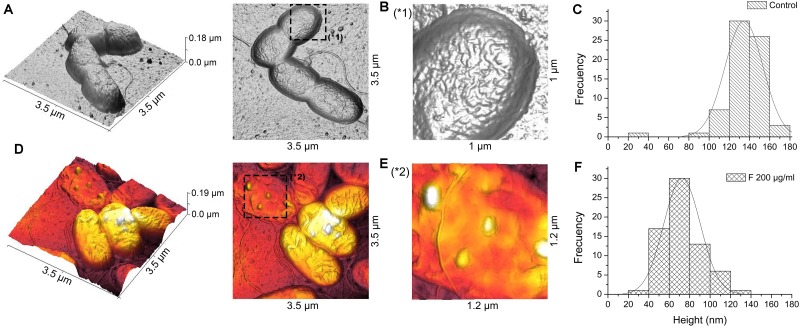
Atomic force microscopy images of *P. aeruginosa* PA01 cells after fengycins exposure. Topography of untreated cells **(A)**. Cells topography after the treatment with 200 μg mL^–1^ of fengycins for 6 h **(D)**. The star symbols (^∗^) reveal zoom area of the figures **(A,B^∗^1)** and **(D,E^∗^2)**. Fengycins treatment resulted in a significant decrease (*p* ≤ 0.05) in height (across the whole cell) of 70 individual PA01 cells in comparison with the control **(C,F)**.

PA01 cells treated with 200 μg mL^–1^ of fengycins during 6 h exhibited reproducible alterations in the cellular topography compared with the control cells (Figure 7). The same alterations reported above for the Xav cells were also observed, to a lesser extent, in PA01 cells; even when the highest concentration of fengycins was applied. In general, following fengycins treatments, PA01 cell surface appeared to lose uniformity exhibiting an altered topography (Figures 7D,E). In contrast, the topography of the untreated PA01 cells appeared far more uniform (Figures 7A,B).

Histograms (Figures 7C,F) of the average height of the PA01 cells exposed to fengycins (200 μg mL^–1^) showed a significant height reduction (72 nm) compared with that of the control cells (136 nm). The high frequencies of height changed from 120 to 160 nm in the untreated PA01 cells to 40–100 nm in the cells exposed to fengycins (Figures 7C,F). The above-mentioned changes suggest that the same cell-perturbing effect observed for Xav occurred.

## Discussion

Morpho-structural alterations of the cell surface and membrane perturbations of several fungal pathogens have been attributed to the effect of CLP (Horn et al., 2013; Wise et al., 2014; Sur et al., 2018; Mantil et al., 2019). Although antifungal activity of CLP has been widely reported (Cawoy et al., 2015), there is less evidence about the effect of these antimicrobials on bacteria. Some authors have attributed to surfactins a moderate antibacterial activity against some pathogenic bacteria, including *S. aureus, B. subtilis, S. typhimurium, P. aeruginosa, C. variabilis*, and *Acinetobacter* (Zhao et al., 2018). Iturins have also been indicated as antibacterial compounds. For example, it was reported that *B. subtilis* SSE4 secreted iturin3 and a new class of CLP from the iturin family, subtulene A. Both CLP showed antibacterial activity against *Stenotrophomonas maltophilia, Acinetobactercal coaceticus, E. coli, S. typhimurium, Enterobacter cloacae*, and *X. campestris* (Thasana et al., 2010).

Although fengycins were best described as antifungal compounds active against plant and human pathogenic fungi we previously reported a strong antibacterial activity of fengycins from MEP_2_18 against Xav. In addition, we suggested that fengycins could impair the biofilm formation and even disrupt those preformed Xav biofilms (Medeot et al., 2017).

This work addresses the study of the effect of fengycins produced by *B*. *amyloliquefaciens* MEP_2_18 on Xav and PA01, a plant pathogen and an opportunistic human pathogen, respectively. A more detailed mass spectrometry analysis revealed that the fraction containing fengycins, previously characterized as fengycins A and B, was indeed a mixture of C16-C17 fengycins isoforms. The antibacterial activity of fengycins on Xav and PA01 was evidenced initially by the presence of inhibition haloes on Petri dishes assays.

The AFM is a powerful tool for depicting the three-dimensional topography of biological samples, including bacteria. The technique allows the characterization of the bacterial cell surface producing high resolution topographical imaging with minimal sample disruption (Müller and Dufrêne, 2011). In this way, AFM experiments performed in this work allowed to obtain topographical and morphological information of changes in the bacterial cell after exposure to fengycins. Untreated Xav cells presented a typical rod shape with a uniform surface topography, which are in agreement with the previously described topography for Xav (Fernandez et al., 2017). At high resolution, alterations on the bacterial surface were observed in Xav and PA01 cells exposed to fengycins. The strength of AFM to visualize alterations at the cell surface level allowed the exploration of the mode of action of several antimicrobial agents (Braga and Ricci, 2011; Dufrêne, 2014; Longo and Kasas, 2014; Fernandez et al., 2017). A decrease in cell height, possibly due to the leakage of intracellular content, was observed. Measurements of potassium efflux confirmed leakage of the intracellular content in Xav cells exposed to fengycins. These data suggest that topographical changes in the cell surface occurred as consequence of the fengycin treatment.

It is known that the lipid composition of the biological membranes and the formation of lipid domains are behind their specific structural properties and functionalities. Bacteria vary widely in the lipid composition of their membranes and would, therefore, be expected to exhibit different sensitivities to antimicrobial compounds that act at the cell surface. For example, works from Epand demonstrated a strong correlation between the lipid composition of bacteria and the ability of antimicrobial agents to induce their toxic effects (Epand and Epand, 2009; Epand et al., 2016). To our knowledge, there are only a few studies addressing the mechanisms of action of the fengycins, and most of them were carried out in very simple membrane models that do not represent the complexity of the biological membranes (Deleu et al., 2005, 2008; Eeman et al., 2005; Fiedler and Heerklotz, 2015). Although the mode of action of the fengycins is not yet understood, our results provide new evidence supporting the role of fengycins as a macromolecule that causes damage to the cellular envelope of sensitive cells.

Molecular dynamics simulations have been used to explain the functional selectivity of fengycins on artificial membranes composed of PE:PG or PC as models for bacterial and eukaryotic membranes, respectively (Sur et al., 2018). Fengycin interacts with lipid monolayers at the air-aqueous interface. It is thought that the ability of fengycins to damage cell membranes, and thereof to cause cell disruption, depends on the aggregation of fengycins and this phenomenon depends on the membrane lipid composition of the target cell. For instance, Sur et al. (2018) demonstrated clear differences in the number, size and lifetime of the fengycin aggregates between both models of membranes, eukaryotic and bacterial. Similarly, Deleu et al. (2008) demonstrated that fengycin insertion into biological membranes produces perturbation and partial dissolution of the dipalmitoylphosphatidylcholine (DPPC) condensed domains, which are partially mixed with fengycin. Those results suggested that fengycin and DPPC interact in the membrane. In agreement with the above mentioned, membranes of both Xav and PA01 strains used in this study contain PC, a phospholipid relatively unusual in bacteria (Geiger et al., 2003; Aktas and Narberhaus, 2015). Based on those studies and depending on the fengycin concentration, two mechanisms of action were proposed: under low fengycin concentrations, fengycin aggregates causing the formation of pores and the subsequent changes in membrane permeability, whereas, at high concentration, fengycin solubilize the membrane similarly as a detergent (Deleu et al., 2005, 2008). Furthermore, it was reported that in DPPC bilayers, fengycin domains are segregated laterally close to the lipid/water interface causing extensive dehydration of the polar region of the membrane. This behavior suggested that fengycin-rich domains may constitute the sites of membrane permeabilization (González-Jaramillo et al., 2017). Other authors highlighted that the insertion of fengycin into the fungal membrane may be associated with low content in anionic phospholipids, thus reducing the electrostatic repulsion with the negatively charged fengycin (Wise et al., 2014). The differences in sensitivity to fengycins observed in Xav and PA01 could be attributed to the intrinsic membrane lipid composition of these bacterial species.

Moreover, it is well-known that fengycins are fungicides but they have little or no effect on mammal cells, both eukaryotic membranes. It has been speculated that this differential selectivity of fengycins may be attributed to the presence of different sterols, ergosterol in the fungal membranes and cholesterol in the mammalian membranes. Apparently, cholesterol may have a protective effect on mammalian membranes (Fiedler and Heerklotz, 2015).

In summary, the AFM was a useful tool to analyze the alterations in the topography of Xav and PA01 cells in response to the exposure to C16–C17 fengycin isoforms produced by *B*. *amyloliquefaciens* MEP_2_18. Interestingly, in this work, we also showed that the cell viability of human normal lung fibroblasts was not affected after treatment with the highest concentration of fengycins tested. More intriguing, there are reports indicating that fengycins exposure, until 200 μg mL^–1^, may play certain inhibitory roles on the development and progression of cancer colon ephitelial cells whereas no cytotoxic effects have been observed in normal colon ephitelial cells (Yin et al., 2013; Cheng et al., 2016). Despite these reports, there are no studies addressing this selective targeting of the fengycins for cancer cells. The results presented in this work give visual examples and real quantitative data on the poor characterized role of fengycins as antibacterial compounds. Fengycin exposure causes dramatic and unprecedented alterations in the topography of Xav and PA01 cells, which result in the cell death by leaking of the intracellular content.

Considering that the application of antimicrobial peptides in clinical has provided to be highly relevant for the treatment of diseases caused by bacteria the data present here could be a start point for the design of novel antimicrobial agents.

## Data Availability Statement

The datasets generated for this study are available on request to the corresponding author.

## Author Contributions

All authors have seen and approved the content and have contributed significantly to this work. DM and EJ conceived the study and drafted the manuscript with the help from all other co-authors. DM and MF performed the experimental work. GM and EJ contributed to the data analysis.

## Conflict of Interest

The authors declare that the research was conducted in the absence of any commercial or financial relationships that could be construed as a potential conflict of interest.

## Footnotes

1 http://gwyddion.net/

2 http://www.r-project.org/
